# Infrared Visual Sensing Detection of Groove Width for Swing Arc Narrow Gap Welding

**DOI:** 10.3390/s22072555

**Published:** 2022-03-26

**Authors:** Na Su, Jiayou Wang, Guoxiang Xu, Jie Zhu, Yuqing Jiang

**Affiliations:** Jiangsu Provincial Key Laboratory of Advanced Welding Technology, School of Materials Science and Engineering, Jiangsu University of Science and Technology, Zhenjiang 212003, China; sunahao2008@163.com (N.S.); xugxiang@163.com (G.X.); zhujie_5858@163.com (J.Z.); jyuqing2012@126.com (Y.J.)

**Keywords:** narrow gap welding, visual sensing, groove width detection, global pattern recognition, dynamic clustering

## Abstract

To solve the current problem of poor weld formation due to groove width variation in swing arc narrow gap welding, an infrared passive visual sensing detection approach was developed in this work to measure groove width under intense welding interferences. This approach, called global pattern recognition, includes self-adaptive positioning of the ROI window, equal division thresholding and in situ dynamic clustering algorithms. Accordingly, the self-adaptive positioning method filters several of the nearest values of the arc’s highest point of the vertical coordinate and groove’s same-side edge position to determine the origin coordinates of the ROI window; the equal division thresholding algorithm then divides and processes the ROI window image to extract the groove edge and forms a raw data distribution of groove width in the data window. The in situ dynamic clustering algorithm dynamically classifies the preprocessed data in situ and finally detects the value of the groove width from the remaining true data. Experimental results show that the equal division thresholding algorithm can effectively reduce the influences of arc light and welding fume on the extraction of the groove edge. The in situ dynamic clustering algorithm can avoid disturbances from simulated welding spatters with diameters less than 2.19 mm, thus realizing the high-precision detection of the actual groove width and demonstrating stronger environmental adaptability of the proposed global pattern recognition approach.

## 1. Introduction

Narrow gap gas metal arc welding (NG-GMAW) is a high-efficiency and high-quality welding process for manufacturing thick-section structures [1,2,3,4]. To solve the problem of sidewall penetration, several single-wire NG-GMAW processes have been developed. High-speed rotation arc with an eccentric contact tip [5,6], twist wire [7] and wave-shaped wire [8] have been designed to improve the penetration into groove sidewalls. To further increase the practicality of the rotation arc, a rotation arc welding system [9] driven by a hollow axis motor was presented to rotate the arc directly while reducing torch volume. However, rotation arc processes are not suitable for all positions; besides the easy wear of their contact tips, the pre-bending wires cause poor directivity of the arc. To overcome these shortcomings, a swing arc narrow gap welding (SA-NGW) process was developed [10,11,12], which utilizes a hollow axis motor to directly drive a bending conductive rod to circularly swing the arc. Furthermore, three-dimensional numerical models [13,14] were established to analyze the temperature and flow fields for process optimization. This novel process was successfully applied to the horizontal and vertical-up welding at groove widths of 12 to 16 mm.

In narrow gap welding, the groove width and weld central position usually vary due to the groove processing error, assembling error and welding thermal deformation, which leads to uneven sidewall penetrations and inconsistent bead surface [15,16,17]. To avoid poor weld formation due to groove variation, several passive visual sensing detection methods have been proposed [18,19,20,21,22]. Yamazaki et al. [18] used a CMOS camera to capture infrared images of the welding zone and detected the width and central position of the narrow gap laser welding groove using brightness distribution analysis, but it is difficult to adaptively determine a threshold for the gradient of the brightness distribution curve with the occurrence of laser plume and welding spatter. Zhu et al. [19] proposed a local pattern recognition algorithm for the groove edge position to detect the weld deviation from infrared images in the SA-NGW process, but this algorithm is readily trapped in local optima. Li et al. [21] employed an infrared camera to detect the weld deviation for the rotation arc process by calculating the relative distance of the gravity center of the arc to the sidewall to which the arc rotates, without involving spatter and fume disturbances. Chen et al. [22] visually identified the weld deviation for narrow gap K-TIG welding by comparing the weld centerline to the keyhole center; this TIG process usually leads to little spatter. In practice, passive visual sensing approaches are also involved in other welding processes [23,24,25,26,27,28,29,30,31]. Pinto-Lopera et al. [23] applied a CMOS infrared camera to collect images of the molten pool for identifying the width and height of the GMAW process. Shao et al. [26] employed a CCD infrared sensor to obtain welding images and estimated the seam deviation from the images by a particle filtering algorithm for narrow butt-joint laser welding. Xiong et al. [28] utilized a CCD sensor with a 685 nm narrowband filter to acquire clear images of the molten pool and reconstructed the 3D shape of the molten pool by pool geometric features in gas metal arc additive manufacturing. The above visual sensing detection approaches can realize synchronic detection with the arc position without an additional light source. Nevertheless, their adaptivity to welding processes and their capability of resisting welding interferences need to be further improved, particularly for rotating and swing arc narrow gap welding processes, in which welding spatter easily attaches to the groove sidewall in addition to the uniform accumulation of welding fume in the narrow gap groove.

To adaptively control weld formation for the SA-NGW process with variable grooves in the future, an infrared passive visual sensing detection approach and system were developed in the present work to measure groove width under intense welding interferences. This approach detects groove width from disturbed images of welding by global pattern recognition based on the adaptive extraction of the groove edge and the dynamic classification of groove width data. Accordingly, an in situ dynamic clustering algorithm is presented to identify the value of groove width from the classified true data in the data window after accurately extracting the groove edge by an equal division thresholding method. Additionally, simulated welding spatters and actual welding experiments were performed to demonstrate the anti-interference capability and effectiveness of the proposed approach so as to build a foundation for self-adaptive controls of the arc swinging angle and welding deposit in a groove of varying width.

## 2. Infrared Passive Visual Sensing Detection System for Groove Width

### 2.1. System Construction

**Figure 1:**
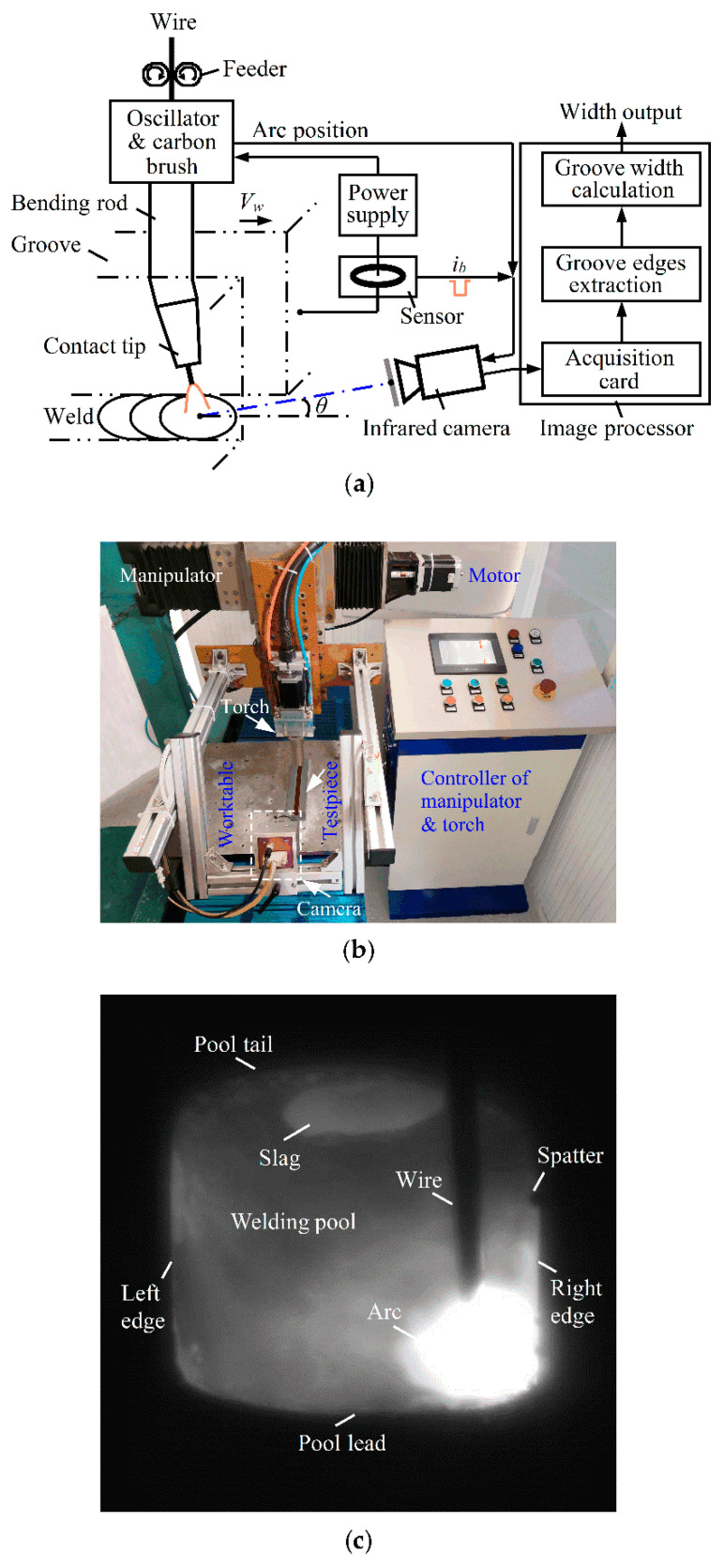
Principle of infrared passive visual sensing detection system of groove width for swing arc narrow gap welding: (**a**) schematic configuration of system; (**b**) actual system photograph; (**c**) example of welding infrared image.

Figure 1 shows the principle of the infrared passive visual sensing detection system of groove width for swing arc narrow gap welding. Figure 1a shows the schematic configuration of this system, which includes a swing arc narrow gap welding torch, welding power supply, Hall current sensor, CMOS camera with infrared narrowband and neutral density filters and computer image processor. The torch consists of an oscillator, carbon brush, bending rod and contact tip. Figure 1b presents an actual photograph of this system. It can be seen from this picture that this system additionally contains the manipulator and the controller of the manipulator and torch. This manipulator comprises three regulating mechanisms: the vertical and horizontal ones adjust the height and transverse position of the torch, and the other one drives the worktable carrying the test piece to move relatively to the torch and thus gives a welding speed of *V_w_*. Note that the test piece is actually fixed to the worktable during welding.

The positive pole of the power supply is connected to the carbon brush in the torch, and the current sensor detects the current signal of the pulsed arc. The camera is fixed to the torch, the lens of which is axially separated from the wire end by a distance of 270 mm and is directed at the welding pool ahead of the torch at a depression angle of *θ*. At lower frequencies of several hertz, the torch oscillator periodically turns the bending conductive rod to swing the arc circularly at the wire tip within the narrow gap groove. When the arc swings to every sidewall of the groove, this oscillator synchronously outputs a signal of the arc position once the arc pauses briefly at the sidewall. This arc position signal and the base current signal *i_b_* of the pulsed arc simultaneously trigger the camera to collect the image of the welding area. After the image is transferred to the acquisition card and processed by the computer, the groove edge on the opposite side of the arc is extracted to avoid arc light interference, and finally, the value of groove width is detected from the left and right edges of the groove.

An example of the actual welding infrared image is shown in Figure 1c when the arc remains near the right sidewall of a constant-width groove, and other images that reflect the significant variations in groove width are shown in Section 5.3 for a width-varying groove. For the test involved in Figure 1c, the experimental conditions for camera shooting and narrow gap pulsed arc welding are listed in Table 1 and Table 2, respectively, and hereafter, the related conditions are the same as those unless otherwise specified. Moreover, to ensure the clarity of the collected images, the focal length of the camera must be fine-tuned for each test, which probably results in a minor difference in the calibrated value of each pixel. This image demonstrates that arc light and welding fume disturbances can be effectively reduced by having the camera capture the welding image at the opposite side of the arc during the base current of the arc. Finally, we can observe clear images of the arc, wire, molten pool, left and right edges of the groove, and welding spatter attached to the groove edge. However, welding fume and arc light still significantly influence the uniformity of image grayscale in the groove area, which, together with welding spatter, raises the difficulty in actual groove edge extraction and width detection.

### 2.2. Detection Principle

**Figure 2:**
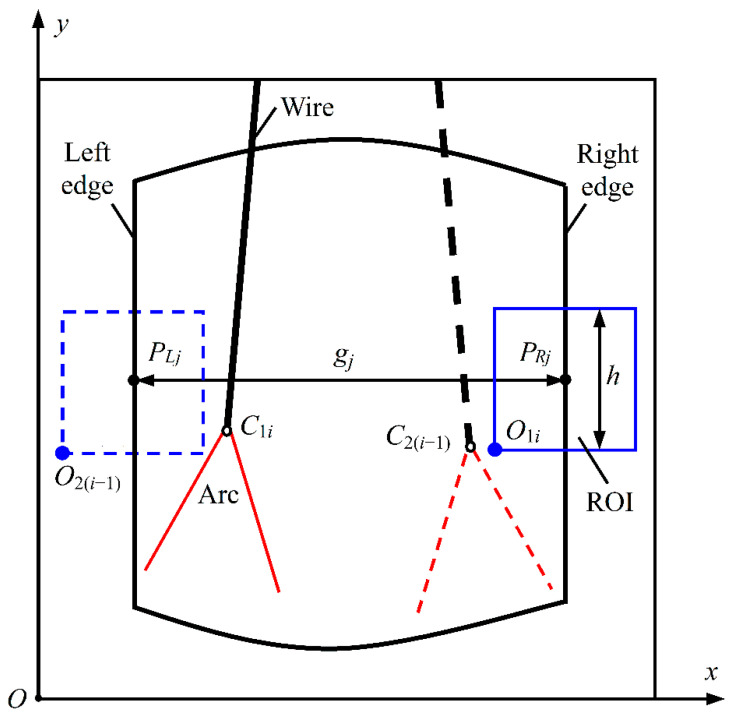
Principle of groove width detection approach for swing arc narrow gap welding.

Figure 2 shows the principle of the groove width detection approach for swing arc narrow gap welding, where *i* indicates the *i*th frame of the welding image, and *O*_1*i*_ and *O*_2(*i*−1)_ are the current and previous origins of the region-of-interest (ROI) window. From the current and previous adjacent frames of ROI window images, i.e., the *i*th and the (*i* − 1)th frames, the positions *P_Rj_* (*x_Rj_*, *y_Rj_*) and *P_Lj_* (*x_Lj_*, *y_Lj_*) of right and left edge points of the groove are extracted, and then the corresponding raw datum $g_{j}$ of groove width within the height range of the ROI window is found by Equation (1).

(1) $$g_{j}{=(x}_{\mathrm{Rj}}\;{-\;x}_{\mathrm{Lj}})\;(0\;\leq \;j\;\leq \;h-1)$$

where *j* is the ordinal number of the position of $g_{j}$, and *h* is the height of the square ROI window and expresses an integer value in pixels. Accordingly, the raw data distribution matrix $G_{i}^{\;r}\;$ of groove width, which contains *h* data, is formed from $g_{j}$. The current sampling value of groove width is determined by using the ISDC algorithm (described later) to remove false data from $G_{i}^{\;r}$.

Since the positions of arc highest point and groove edge actually vary in welding, the position of the groove-edge ROI window needs to be adjusted in real time to overcome such interferences and to accurately intercept the groove edge image on the opposite side of the arc. Accordingly, in image processing, a global image (i.e., welding image) will be processed sequentially through Gaussian filtering, global thresholding and a morphological opening algorithm [32]. The current and previous highest points *C*_1*i*_ (*x*_arc_, *y*_arc_) and *C*_2(*i*−1)_ of the arc profile are then extracted, respectively. To self-adaptively position the ROI window on the global image, five values of *y*_arc_ sampled in the last adjacent five frames of welding images are filtered, and the value of the vertical coordinate *y*_ROI_ for the origin *O*_1*i*_ of the current ROI window is achieved after subtracting a constant *δ* from this filtered result ${\hat{y}}_{\mathrm{arc}}$. Moreover, the value of the horizontal coordinate *x*_ROI_ for this origin *O*_1*i*_ is determined by subtracting the half-width (*h*/2) of the ROI window from the estimated positional value ${\hat{x}}_{i-2}$ of the same-side groove edge on the previous frame of the ROI window image, so the groove edge is always approximately on the central line of the window width. Consequently, the position regulation of the ROI window self-adapts to the arc position, according to Equation (2).

(2) $$\left\{ \begin{array}{l} x_{\mathrm{ROI}}={\hat{x}}_{i-2}-h/2\\ y_{\mathrm{ROI}}={\hat{y}}_{\mathrm{arc}}-\delta \end{array}\right.$$

Furthermore, for a groove edge image intercepted by the ROI window, a groove edge is extracted by using a median filter, contrast stretching, the Otsu thresholding algorithm, a morphological closing algorithm and the Canny edge detector [32] in turn. In this case, because the difference between two adjacent coordinates of *y*_ROI_ lies merely within ±0.3 mm, the value of the groove width can be approximately obtained directly from the left and right groove edges that are extracted from two neighboring frames of ROI window images.

According to the above self-adaptive positioning of the ROI window, together with the following methods of equal division thresholding (EDT) (see Section 3) and in situ dynamic clustering (ISDC) (see Section 4), a global pattern recognition (GPR) approach is formed to accurately detect groove width.

## 3. Division Thresholding Method of ROI Window Image

In SA-NGW, arc light and welding fume uniformly distribute in the welding region because the arc moves in a narrow gap groove, and as a result, the uniformity of image grayscale distribution is greatly affected. Consequently, it is difficult to accurately extract the groove edge using the process of Otsu thresholding segmentation from the whole ROI window image of the groove edge. To improve the extraction accuracy of the groove edge in the window image, an equal division thresholding (EDT) method is proposed. This method vertically divides the ROI window image into several equal partitions and then uses Otsu thresholding to segment each partial image for the purpose of accurately extracting each subsection of the groove edge to form the full edge of the groove.

**Figure 3:**
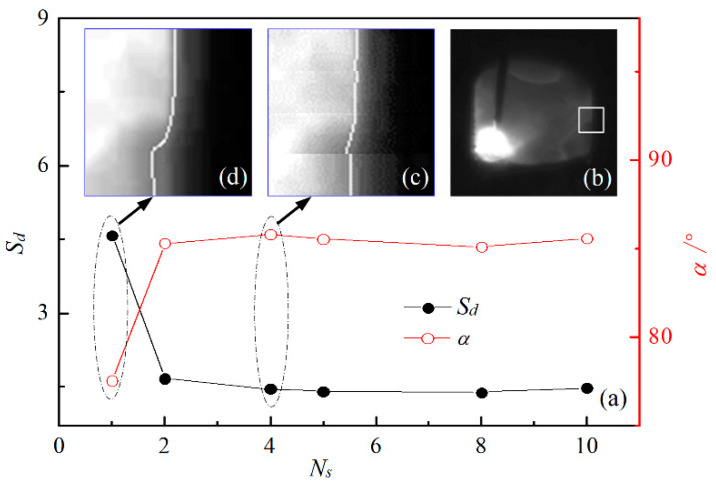
Effect of vertical equal partition number *N_s_* of ROI window image on the extraction accuracy of groove edge (*S_d_* is the standard deviation of positional distribution of groove edge points, and *α* denotes the slope angle of fitting line of groove edge points: (**a**) effect of *N_s_* on *S_d_* and *α*; (**b**) example of ROI window in welding image; (**c**) four partitions of ROI window image; (**d**) single partition of ROI window image.

Figure 3 shows the effect of the vertical equal partition number *N_s_* of the ROI window image on the extraction accuracy of the groove edge, where *S_d_* represents the standard deviation of the positional distribution of groove edge points, and *α* denotes the slope angle of the fitting line of groove edge points. Figure 3a shows the influence of the partition number *N_s_* on the standard deviation *S_d_* and the slope angle *α*, where the used ROI window image is cut from the welding image and indicated by the area in the white box in Figure 3b. As the number *N_s_* of vertical equal partitions increases, the slope angle *α* increases while the standard deviation *S_d_* decreases, and correspondingly, the perpendicularity of the fitting line of the groove edge improves. In particular, when the number *N_s_* of partitions is greater than 4, the variations in *α* and *S_d_* become gentle, which indicates that the extracted horizontal positions of groove edge points change little and thus suggests that the extraction accuracy of the groove edge becomes high.

For example, due to the influence of arc light and welding fume, when the number *N_s_* of partitions is 1, the lower partition of the groove edge contour line extracted after whole-threshold processing develops a pronounced bend, as shown by the white line in Figure 3d. In this case, the slope angle *α* is the smallest, while the standard deviation *S_d_* is the largest, which implies that the positional dispersity of groove edge points is the largest. When the number *N_s_* of partitions is 4, the whole ROI window image is equally cut into four partitions, whose dividing lines are represented by yellow dashed lines in Figure 3c; each partition of the groove edge is extracted separately from each corresponding partial image, as shown by the white line in Figure 3c. Accordingly, *α* increases from 77.55° to 85.79°, while *S_d_* decreases from 4.581 to 1.449, and the verticality of the fitting line of the groove edge improves. Compared to the whole-thresholding approach, the division thresholding method with four equal partitions reduces the positional dispersity of groove edge points by ~68.37%, which indicates a significant improvement in the verticality of the groove edge fitting line. Therefore, the vertical equal partition number *N_s_* of the ROI window image is taken as 4.

## 4. In Situ Dynamic Clustering Algorithm

The principle of the in situ dynamic clustering (ISDC) algorithm proposed is introduced below through flow charts and mathematical expressions. Dynamic analysis of this algorithm is then presented for intuitive understanding. Subsequently, the adaptivity of this algorithm is investigated by simulated spatters to demonstrate the capability of resisting welding spatter disturbance.

### 4.1. Principle of ISDC Algorithm

**Figure 4:**
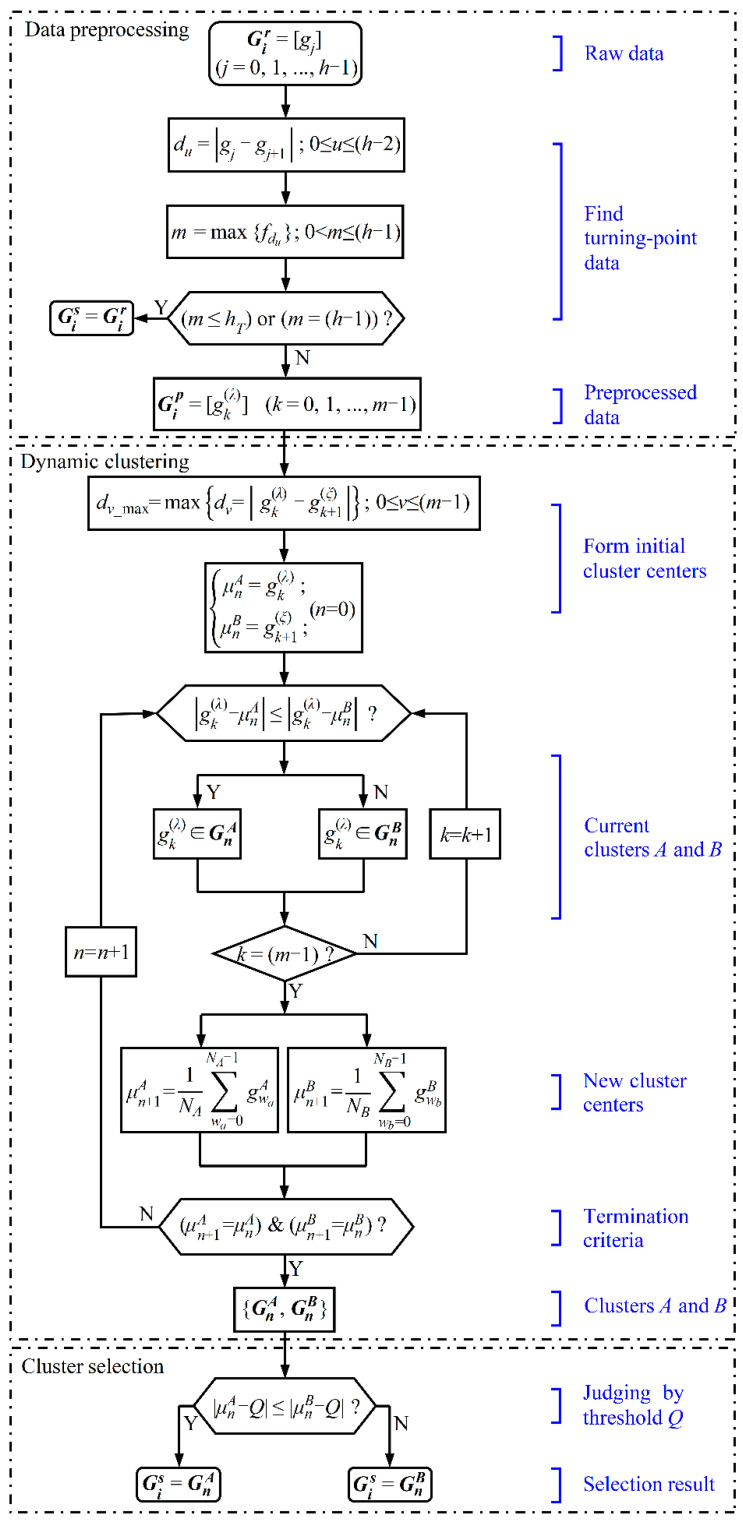
Schematic diagram of the ISDC algorithm.

To reduce the influence of welding spatter on the accuracies of groove edge extraction and groove width detection, the ISDC algorithm is proposed for detecting groove width. This algorithm performs an in situ global dynamic bipartite clustering of raw groove width data in the data window according to the numerical difference degree and removes false data from the raw data according to outlier criteria, finally utilizing linear fitting or mean calculation to obtain the sampling value of the groove width from the preserved true data in the data window. The algorithm process includes data preprocessing, dynamic clustering and cluster selection, as shown in Figure 4.

#### 4.1.1. Data Preprocessing

For the purpose of eliminating the turning-point data in the raw data distribution of groove width and thus improving data separability, a data preprocessing algorithm based on the numerical difference degree between adjacent values is presented. The raw datum $g_{j}$ of groove width is arranged in order of its position along the height direction of the ROI window, finally forming a raw data distribution matrix $G_{i}^{\;r}{=[g}_{j}^{\;}]$ of groove width. The degree of numerical difference between adjacent data in $G_{i}^{\;r}$ is then evaluated by the absolute difference *d_u_* in Equation (3).

(3) $$d_{u}^{\;}=\left|g_{j}^{\;}\;{-\;g}_{j+1}^{\;}\right|\;(0\;\leq \;u\;\leq \;h-2)$$

where *u* is the ordinal number of the position of *d_u_*. The frequency *f_du_* at which different values of *d_u_* appear is then counted to determine its greatest value *m*$\;=\max\left\{f_{d_{u}}\right\}$ and the numerical difference degree of $d_{u}^{\;m}$ corresponding to this maximum. Subsequently, the data satisfying the condition of $d_{u}^{\;}\;$=${\;d}_{u}^{\;m}$ from the raw data distribution matrix $G_{i}^{\;r}$ are selected to build a preprocessed data distribution matrix $G_{i}^{\;p}$ of groove width according to their corresponding sequences in $G_{i}^{\;r}$, as shown in Equation (4).

(4) $$G_{i}^{\;p}{=[g}_{k_{\;}^{\;}}^{(\lambda )}]\;(k=0,\;1,\;\ldots {,\;m-1)\;}_{\;}^{\;}$$

where $g_{k_{\;}^{\;}}^{(\lambda )}$ represents any of the preprocessed groove width data; *k* is the ordinal number of the current position of $g_{k_{\;}^{\;}}^{(\lambda )}$ in $G_{i}^{\;p}\;$, and *λ* denotes the ordinal number of the original position of $g_{k_{\;}^{\;}}^{(\lambda )}$ in $G_{i}^{\;r}$.

When all raw width data are of the same value, or when the number of remaining data is less than a certain value $h_{T}$, that is, for the case of *m* = (*h*−1) or *m* ≤ *h_T_*, respectively, the raw groove width data are directly used as true data (i.e., $G_{i}^{\;s}{=G}_{i}^{\;r}$); otherwise, true data are chosen by the following dynamic clustering. In our experiments, *h_T_* was selected as 10% × *h* so as to reduce the influence of the small quantity of true data on the detection accuracy of groove width.

#### 4.1.2. Dynamic Clustering

##### Determination of Initial Cluster Center

For the preprocessed data distribution matrix $G_{i}^{\;p}$ of groove width, the numerical difference degree *d_v_* of every two data at adjacent positions is calculated, and then the maximum value *d_v__*_max_ of all degrees is determined according to Equation (5). The two data $g_{k_{\;}^{\;}}^{(\lambda )}$ and $g_{{k+1}_{\;}^{\;}}^{(\xi )}$ corresponding to *d_v__*_max_ are respectively used as the initial centers of two clusters, as shown in Equation (6).

(5) $$d_{v\_\max}=\max\left\{d_{v}^{\;}=\left|{\;g}_{k_{\;}^{\;}}^{(\lambda )}{\;-\;g}_{{k+1}_{\;}^{\;}}^{(\xi )}\right|\right\};\;0\;\leq \;v\leq \;(m-1)$$

(6) $$\{\begin{array}{c} \mu_{n}^{A}\;{=g}_{k_{\;}^{\;}}^{(\lambda )};\\ \mu_{n}^{B}\;{=g}_{k+1}^{(\xi )}; \end{array}\;(n=0)$$

where *ξ* denotes the ordinal number of the original position of $g_{k+1}^{(\xi )}$ in $G_{i}^{\;r}$; $\mu_{n}^{A}$ and $\mu_{n}^{B}$ are the initial cluster centers of *A*-cluster data distribution matrix $G_{n}^{A}$ and *B*-cluster data distribution matrix $G_{n}^{B}$, respectively; and *n* is the iteration number of dynamic clustering.

##### Dynamic Clustering

According to the numerical difference degree, which is represented by the absolute difference between each datum in $G_{i}^{\;p}$ and one ($\mu_{n}^{A}$ or $\mu_{n}^{B}$) of two cluster centers, the global dynamic bipartite clustering of $G_{i}^{\;p}$ is carried out. If Equation (7) is satisfied, which indicates that the degree ($\left|g_{k_{\;}^{\;}}^{(\lambda )}\;{-\;\mu }_{n}^{A}\right|$) of the numerical difference between datum $g_{k_{\;}^{\;}}^{(\lambda )}$ and cluster center $\mu_{n}^{A}$ is relatively small, datum${\;g}_{k_{\;}^{\;}}^{(\lambda )}$ is classified in situ into cluster $G_{n}^{A}$, i.e.,${\;g}_{k_{\;}^{\;}}^{(\lambda )}\;\in {\;G}_{n}^{A}$; otherwise, there exists a smaller degree ($\left|g_{k_{\;}^{\;}}^{(\lambda )}\;-\mu_{n}^{B}\right|$) of numerical difference between datum ${\;g}_{k_{\;}^{\;}}^{(\lambda )}$ and cluster center $\mu_{n}^{B}$, and datum $g_{k_{\;}^{\;}}^{(\lambda )}$ is accordingly sorted in situ into cluster $G_{n}^{B}$, i.e., ${\;g}_{k_{\;}^{\;}}^{(\lambda )}\;\in {\;G}_{n}^{B}$. The above process is not completed until all data in $G_{i}^{\;p}$ are classified. Consequently, the in situ dynamic clustering of data is realized by retaining the ordinal number *λ* of the original position of $g_{k_{\;}^{\;}}^{(\lambda )}$ in $G_{i}^{\;r}$.

(7) $$\left|g_{k_{\;}^{\;}}^{(\lambda )}{-\;\mu }_{n}^{A}\right|\;\leq \;\left|g_{k_{\;}^{\;}}^{(\lambda )}{\;-\;\mu }_{n}^{B}\right|$$

##### Renewal of Cluster Center

The centers of clusters $G_{n}^{A}$ and $G_{n}^{B}$ are recalculated by Equation (8), and then it is judged whether the values of the two new cluster centers ($\mu_{n+1}^{A}$ and $\mu_{n+1}^{B}$) equal the values of the previous two cluster centers ($\mu_{n}^{A}$ and $\mu_{n}^{B}$), respectively, or not. If not, $\mu_{n+1}^{A}$ and $\mu_{n+1}^{B}$ are updated accordingly as the cluster centers of $G_{n}^{A}$ and$\;G_{n}^{B}$, and the above dynamic classification operation is repeated and not completed until the updated cluster centers ($\mu_{n+1}^{A}$ and $\mu_{n+1}^{B}$) are respectively equal to the previous two; otherwise, the bipartite clustering operation of $G_{i}^{\;p}$ ends. Consequently, $G_{i}^{\;p}$ is divided into two stable clusters, namely,$\;G_{n}^{A}$ and $G_{n}^{B}$.

(8) {μn+1A=1NA∑wa=0NA-1gwaA μn+1B=1NB∑wb=0NB-1gwbB

where *w_a_* and *w_b_* are the positional variables of the data in $G_{n}^{A}$ and $G_{n}^{B}$, $g_{w_{a}}^{A}$ and $g_{w_{b}}^{B}$ denote the values of groove width in $G_{n}^{A}$ and $G_{n}^{B}$, and *N_A_* and *N_B_* are the total numbers of data in $G_{n}^{A}$ and $G_{n}^{B}$, respectively.

#### 4.1.3. Cluster Selection

By sequencing the data in the matrix $G_{i}^{\;r}$ of groove width from the smallest to the largest, the corresponding value of groove width at a certain position quantile is taken as the threshold *Q* of cluster selection. By calculating the numerical difference degree between the two cluster centers ($\mu_{n}^{A}$ and $\mu_{n}^{B}$) and *Q*, the true data are selected in accordance with the outlier criteria in Equation (9).

(9) $$\left|\mu_{n}^{A}-\;Q\right|\;\leq \;\left|\mu_{n}^{B}\;-\;Q\right|$$

where the value of *Q* is determined by subsequent experiments in Section 4.3.1. If Equation (9) holds, $G_{n}^{A}$ is chosen as the true data distribution matrix $G_{i}^{\;s}$ (i.e., $G_{i}^{\;s}{=G}_{n}^{A}$), while $G_{n}^{B}$ is regarded as a false data distribution matrix; otherwise, $G_{n}^{B}$ is used as the true data distribution matrix $G_{i}^{\;s}$ (i.e., $G_{i}^{\;s}{=G}_{n}^{B}$), whereas $G_{n}^{A}$ is regarded as the other one.

### 4.2. Dynamic Analysis of ISDC Algorithm

**Figure 5:**
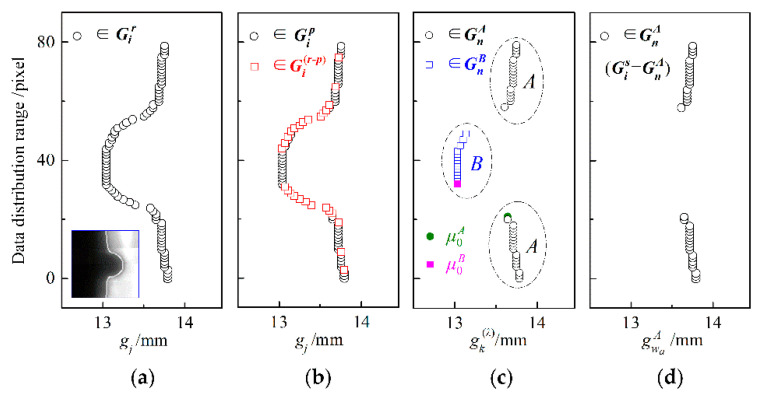
Dynamic solution procedure of the ISDC algorithm: (**a**) distribution of raw width data; (**b**) data preprocessing; (**c**) dynamic clustering; (**d**) cluster selection.

As an example, Figure 5 illustrates the dynamic solution procedure of the ISDC algorithm for a groove of constant width, where the related data distributions at different steps are displayed in the same data window. The height of this data window is the same as that of the ROI window and indicates the distribution range of the data, while the width of this data window reflects the varying range of the groove width. When welding spatter occurs, as shown the ROI image in Figure 5a, the middle segment of the left groove edge bends to the right in the window image. This accordingly causes the left bending of the raw data distribution of groove width in the data window, because each raw datum $g_{j}$ of groove width is obtained by Equation (1) from locations of the left and adjacent right groove edge points, as shown in Figure 5a. Consequently, the raw data distribution matrix $G_{i}^{\;r}$ of groove width is formed, that is, $G_{i}^{\;r}{=[g}_{j}^{\;}]$.

By calculating the degree *d_u_* (in Equation (3)) of the numerical difference between each datum $g_{j}$ and its adjacent one in raw data distribution matrix $G_{i}^{\;r}$, the preprocessed data distribution matrix $G_{i}^{\;p}$ of groove width is obtained after excluding the data in the matrix $G_{i}^{\;(r-p)}$ of turning-point data, where the distributions of data in $G_{i}^{\;p}$ and $G_{i}^{\;(r-p)}$ are exhibited in Figure 5b. Subsequently, after finding the maximum value *d_v_*__max_ (in Equation (5)) of numerical difference degrees between each datum $g_{k_{\;}^{\;}}^{\left(\lambda \right)}$ and its adjacent one in the preprocessed data distribution matrix $G_{i}^{\;p}$, the initial centers ($\mu_{0}^{A}$ and $\mu_{0}^{B}$) of two clusters are determined by Equation (6). Based on the iterative algorithm in Equations (7) and (8) of global dynamic clustering, the two clusters of data distribution matrices ($G_{n}^{A}$ and $G_{n}^{B}$) are then formed, the data of which are also plotted in the data window, as shown in Figure 5c.

Finally, the corresponding raw datum of groove width at the position quantile of 3/4 (i.e., 3/4-quartile) is used as the value of *Q*. After eliminating the false data distribution matrix $G_{n}^{B}$, each $g_{w_{a}}^{A}$ of true data is obtained to constitute the true data distribution matrix $G_{i}^{\;s}$ according to the outlier criteria in Equation (9). The data in $G_{i}^{\;s}$ are visualized in Figure 5d. It is seen from this graph that $G_{i}^{\;s}$ can reasonably characterize the actual data distribution of groove width in the data window.

### 4.3. Adaptability of ISDC Algorithm

#### 4.3.1. Effect of Cluster Selection Threshold

**Figure 6:**
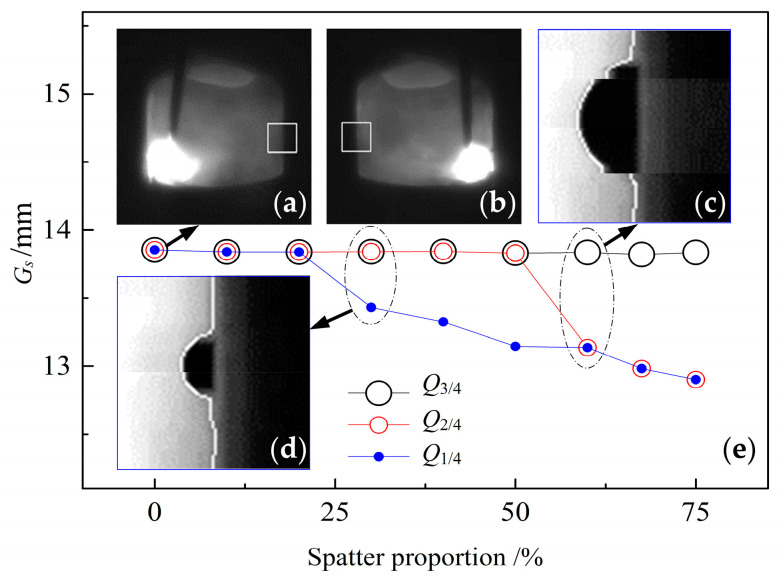
Effect of spatter size proportion and cluster selection threshold *Q* on sampled value *G_s_* of groove width: (**a**) example of welding image for arc at left; (**b**) example of welding image for arc at right; (**c**) simulated spatter of size proportion 60%; (**d**) simulated spatter of size proportion 30%; (**e**) *G_s_* at various *Q* and spatter proportions.

The effect of the cluster selection threshold *Q* on the adaptability of the ISDC algorithm was investigated with the occurrence of simulated welding spatters of different sizes, as shown in Figure 6, where the size proportion of simulated spatter is the ratio of the spatter diameter to the height *h* of the ROI window. Figure 6a,b show two adjacent frames of welding images.

During image processing, the right edge image of the groove is cut by the ROI window from the welding image shown in Figure 6a. The diametrical line of semi-circular simulated spatter is located on the groove edge line in the ROI window image, and then the right groove edge containing the spatter profile is extracted. For size proportions of simulated spatter of 60% and 30%, the extracted profile lines of the right groove edge are indicated as white lines in Figure 6c,d, respectively. The left groove edge is taken by intercepting the left edge image of the groove from Figure 6b through the ROI window in order to form a raw data distribution matrix $G_{i}^{\;r}$ of groove width. Finally, the ISDC algorithm selects the true data distribution matrix $G_{i}^{\;s}$ from $G_{i}^{\;r}$. In this case, if the number of data in $G_{i}^{\;s}$ is greater than 10% × *h*, a linear fitting method is used to find the sampling value *G_s_* of groove width from $G_{i}^{\;s}$, where *G_s_* is derived from the 40th value of *h* (*h* = 80) data on the fitting line of groove width; otherwise, this value of *G_s_* is approximately calculated by finding the mean value of data in $G_{i}^{\;s}$.

Figure 6e represents the effect of the cluster selection threshold *Q* on the sampling value *G_s_* of groove width, where *Q*_1/4_, *Q*_2/4_ and *Q*_3/4_ denote the corresponding values of *Q* at the position quantiles of 1/4, 2/4 and 3/4, respectively, and the method of determining the three values of *Q* is as described in Section 4.1.3. If the size proportion of simulated spatter ranges from 0 to 20%, the three thresholds of *Q* do not influence *G_s_*. When this proportion varies within the range of 20% to 50%, the two thresholds *Q*_2/4_ and *Q*_3/4_ have no effect on *G_s_*. When further increasing it to 75%, the threshold *Q*_3/4_ only slightly affects *G_s_*. In other words, with an increase in the threshold *Q*, the sensitivity of the ISDC algorithm to welding spatter size decreases, which demonstrates that the ISDC algorithm has the ability to increase resistance to welding spatter interference and thus better adapts to welding environments. Therefore, *Q*_3/4_ was chosen as the threshold *Q* in subsequent experiments.

#### 4.3.2. Effect of Welding Spatter

**Figure 7:**
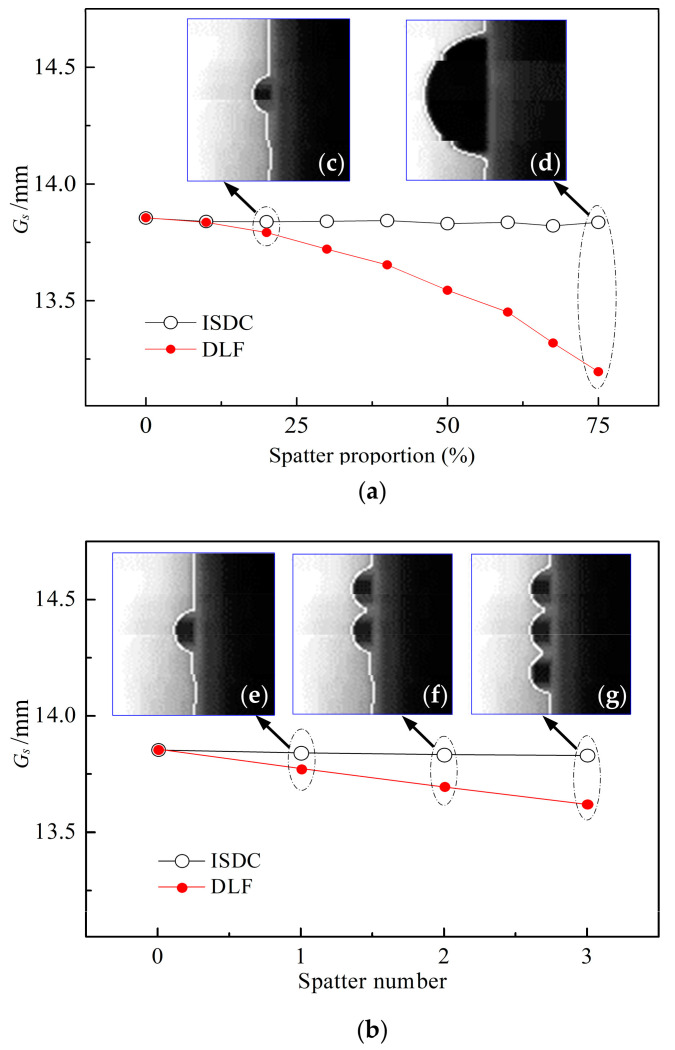
Effect of simulated spatter size proportion and number on sampling value *G_s_* of groove width: (**a**) spatter size proportion; (**b**) spatter number; (**c**) simulated spatter of size proportion 20%; (**d**) simulated spatter of size proportion 75%; (**e**) one simulated spatter in ROI window image; (**f**) two simulated spatters in ROI window image; (**g**) three simulated spatters in ROI window image.

Figure 7 compares the influences of the ISDC algorithm and the direct linear fitting (DLF) method on the sampling values of groove width with different size proportions and numbers of simulated spatters at the chosen threshold *Q*_3/4_ of cluster selection. In this case, each raw data distribution matrix $G_{i}^{\;r}$ and the ISDC-based sampling value *G_s_* of groove width are obtained in the same ways as those in Figure 6. For the DLF method, the sampling value *G_s_* of groove width is obtained by the direct linear fitting of raw groove width data and comes from the 40th value of *h* (*h* = 80) data on the fitting line of groove width.

Figure 7a shows the effects of the size proportion of simulated welding spatter on the sampling value of groove width by using the above two algorithms. For size proportions of 20% and 75%, which correspond to spatter diameters of 0.58 mm and 2.19 mm, the extracted contour lines of the right groove edge are indicated by white lines in Figure 7c,d, respectively. Figure 7b compares the effects of the spatter number on the sampling values of groove width by the two algorithms. Accordingly, the extracted contour lines of the right groove edge are indicated as white lines in Figure 7e–g, where the number of simulated spatters is 1, 2 and 3, respectively.

When increasing this size proportion from 0 to 75%, as shown in Figure 7a, the sampling value *G_s_* of groove width slightly varies with a maximum fluctuation of only 0.034 mm for the ISDC algorithm, while *G_s_* decreases significantly for the DLF method, with a maximum reduction value of 0.659 mm. When increasing the number of spatters from 0 to 3, as indicated in Figure 7b at the per-spatter size proportion of 22.5%, the maximum variation in *G_s_* is merely 0.024 mm for the ISDC algorithm, while *G_s_* decreases markedly in the DLF method, with a maximum decrease of 0.235 mm. Clearly, compared to the DLF method, the ISDC algorithm can stably obtain the sampling value *G_s_* of groove width despite great variation in the number and size of welding spatters, thus indicating the good adaptability of the ISDC algorithm to welding environments.

## 5. Experimental Results of Groove Width Detection

To demonstrate the effectiveness of the GPR approach, several detection experiments of groove width were carried out for a flat-position SA-NGW process under the conditions of constant-width and width-varying grooves. The detection accuracy of the GPR approach was then compared to that of the composite linear fitting (CLF) method, which sequentially combines existing image processing, direct linear fitting and mean filtering algorithms, so as to exhibit the superiority of the proposed approach. Finally, the actual width of the post-welding groove is utilized to validate the suitability of the accuracy evaluation approach.

### 5.1. Experimental Welding Conditions

**Figure 8:**
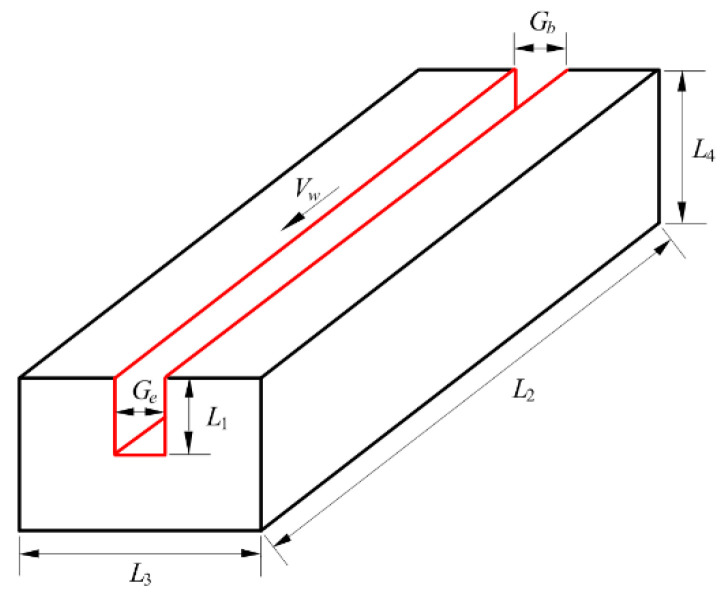
Illustration of test piece.

The experimental conditions are the same as those related to Figure 1c, except for groove width and arc swing angle for a groove of varying width. A test piece with a square groove was machined directly from a mild steel plate with a size of *L*_2_ × *L*_3_ × *L*_4_ (length × width × height), as illustrated in Figure 8, where *G_b_* and *G_e_* are the beginning and ending widths of the groove, respectively, and groove depth *L*_1_ is set as half of *L*_4_. For the experiment with a groove of constant width, the test piece size was 210 × 50 × 40 mm, and the arc swing angle was 82°, while the values of *G_b_* and *G_e_* were 14 mm before welding. For the experiment with a groove of varying width, the test piece size was 204 × 70 × 30 mm, and the arc swing angle was accordingly reduced to 64° due to the smaller starting value *G_b_* of groove width, while the groove width before welding varied uniformly from 11.8 to 15.9 mm.

### 5.2. Experimental Results for Constant-Width Groove

**Figure 9:**
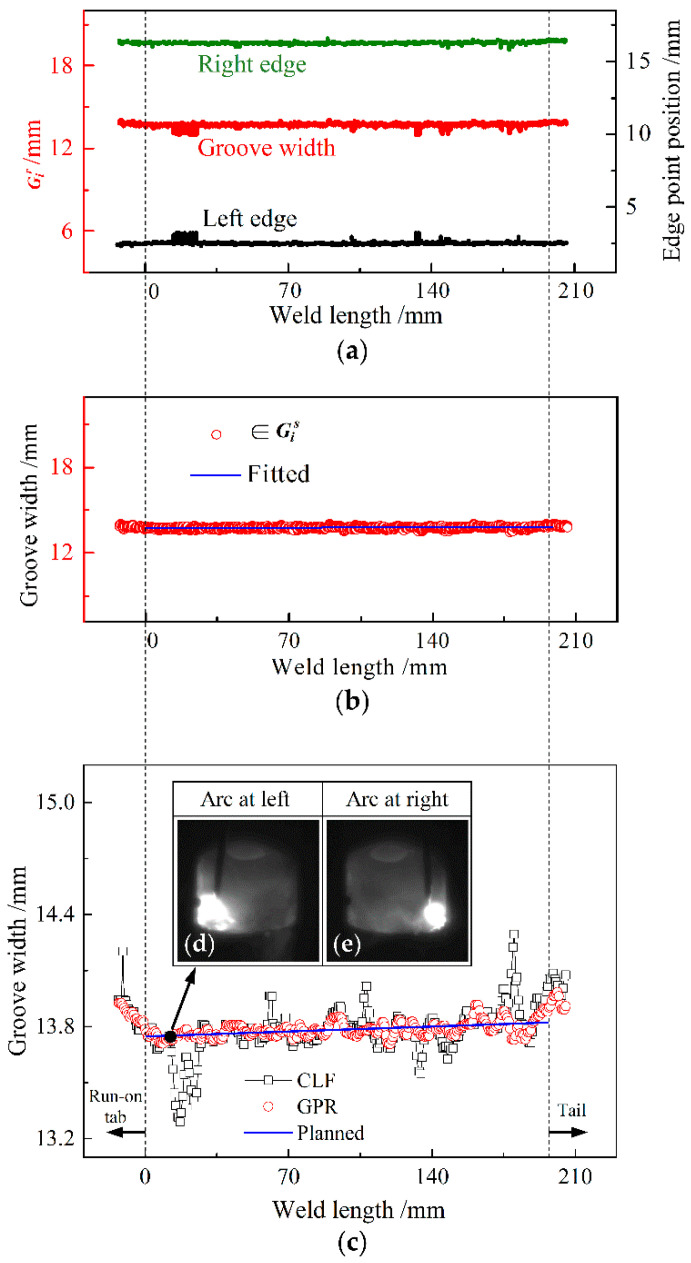
Detected results of constant-width groove: (**a**) edge point positions and instantaneous groove widths containing 322 sets of $G_{i}^{\;r}$; (**b**) true data belonging to $G_{i}^{\;s}$ and fitting line of the true data; (**c**) comparison of detected groove-width values with the planned ones; (**d**) example of global image for arc at left; (**e**) example of global image for arc at right.

Figure 9 shows the detected results for a groove of constant width. During the test, an arc started to ignite on the run-on tab about 13 mm away from the beginning of the groove, and the welding image was collected as the molten pool was stably formed. The arc was extinguished at a distance of about 5 mm from the end of the groove while terminating the collection of welding images. In Figure 9a–c, the dashed line on the left side indicates the boundary line between the run-on tab and the start of the groove; the dashed line on the right side denotes the boundary line of the weld tail, after which the vertical end edges of the groove begin to enter the selecting region of the ROI window image. The locations of these dashed boundary lines are determined by the pre-welding setting and the post-welding observation of welding image characteristics.

During welding, a global image was captured during the swinging of the arc to remain near each groove sidewall, and finally, 323 frames of global images were continuously collected along the whole weld length. By alternately using the ROI window, 162 and 161 frames of left and right groove edge images, respectively, were intercepted at the opposite side of the stationary arc, where one of the left edge images neighbors its previous or subsequent nearest right edge image. Subsequently, 80 instantaneous sampling positional values of corresponding groove edge points were obtained from each edge image to constitute each $G_{i}^{\;r}$, and two distribution graphs of all sampling values from these edge images are plotted for the left and right edge points of the groove by black and green solid points in Figure 9a, respectively. As a result, 322 raw data distribution matrices $G_{i}^{\;r}$ of groove width are formed, where each positional value of a right edge point subtracts each corresponding positional value of the left edge point extracted from two left edge images neighboring the right edge ones, as indicated by red solid points in Figure 9a. Moreover, from the window images, the occurrence of welding spatters is identified at distances of about 14.28 mm, 102.00 mm, 133.28 mm, 147.56 mm and 173.40 mm from the beginning of the groove, and these spatters therefore result in lower instantaneous sampling values of groove width.

Accordingly, 322 sets of $G_{i}^{\;s}$ form along the direction of the weld length after the ISDC algorithm selects the true data distribution matrix $G_{i}^{\;s}$ from each $G_{i}^{\;r}$. After excluding the corresponding data to the two transiting periods (outside of the dashed boundary lines) of welding from 322 sets of $G_{i}^{\;s}$, the remaining true data, comprising 290 sets of $G_{i}^{\;s}$, are linearly fitted along the weld length, and these data together with their fitting line are indicated by red hollow circles and the blue line in Figure 9b, respectively. The fitting line is obtained from a large number of the remaining data and can thus accurately reflect the dynamic changes in the actual groove width during welding. Because of its instantaneous characteristics and statistical features, this line is approximated as the real-time baseline (the same bellow) for evaluating the detection accuracy of groove width, as indicated in blue in Figure 9b.

Figure 9c compares the detected groove width values with the planned ones, where Figure 9d,e show two neighboring global images at weld lengths of 12.24 mm and 12.92 mm. Using the GPR approach, each sampling value *G_s_* of groove width is generated from each $G_{i}^{\;s}$, and then each detected value *G_d_* of groove width is subsequently obtained by the amplitude-limiting mean filtering of the last five successive values of *G_s_*, as indicated by red hollow circles in Figure 9c. During the above two transiting periods, the arc moves transitionally on the run-on tab, and within the ending segment of the groove, some or all of the groove edges in the ROI window image correspond to the vertical heading/ending edges of groove sidewalls. In these two cases, the vertical edges are closer to the camera, which causes larger detected values of groove width, thus leading to the upward bending at the beginning and ending parts of the distribution plots of detected values, indicated by the red hollow circles and black hollow squares in Figure 9c.

When using the CLF method, 322 sampling values of *G_s_* are calculated by direct linear fitting of 322 sets of $G_{i}^{\;r}$, and then 322 detected values of *G_d_* are obtained after the mean filtering of the last five successive values of *G_s_*, as indicated by black hollow squares in Figure 9c. In this case, the detected values *G_d_* become very low near the positions of the above spatters, particularly around 14.28 mm and 133.28 mm of the weld length. Compared with the CLF method, the GPR approach significantly reduces the influence of welding spatters on the detected values of groove width. Compared with the planned values of groove width on the blue baseline, the detection error of groove width is within −0.086 to +0.109 mm when using the GPR approach and between −0.465 mm and +0.479 mm when using the CLF method. In addition, the standard deviations of the detected values are 0.035 and 0.115 for the two methods, respectively, because thresholding with four equal divisions in the GPR approach has a higher accuracy of groove edge extraction (see example in Figure 3), and the ISDC algorithm in the GPR approach has a stronger capability of resisting welding spatter (see Figure 7). Therefore, the proposed GPR approach can realize high-accuracy width detection for a groove of constant width, regardless of welding disturbances.

The related performance comparison is listed in Table 3, where the standard deviation of the groove edge point positional distribution and the size of resistible spatter come from Figure 3 and Figure 7a, respectively. Since the CLF method excludes equal division thresholding but includes the DLF algorithm, its standard deviation of groove edge point positional distribution corresponds to that of *N_s_* = 1 in Figure 3, and its size of resistible spatter corresponds to that of the DLF algorithm at a spatter size proportion of 20% in Figure 7a.

### 5.3. Experimental Results for Width-Varying Groove

**Figure 10:**
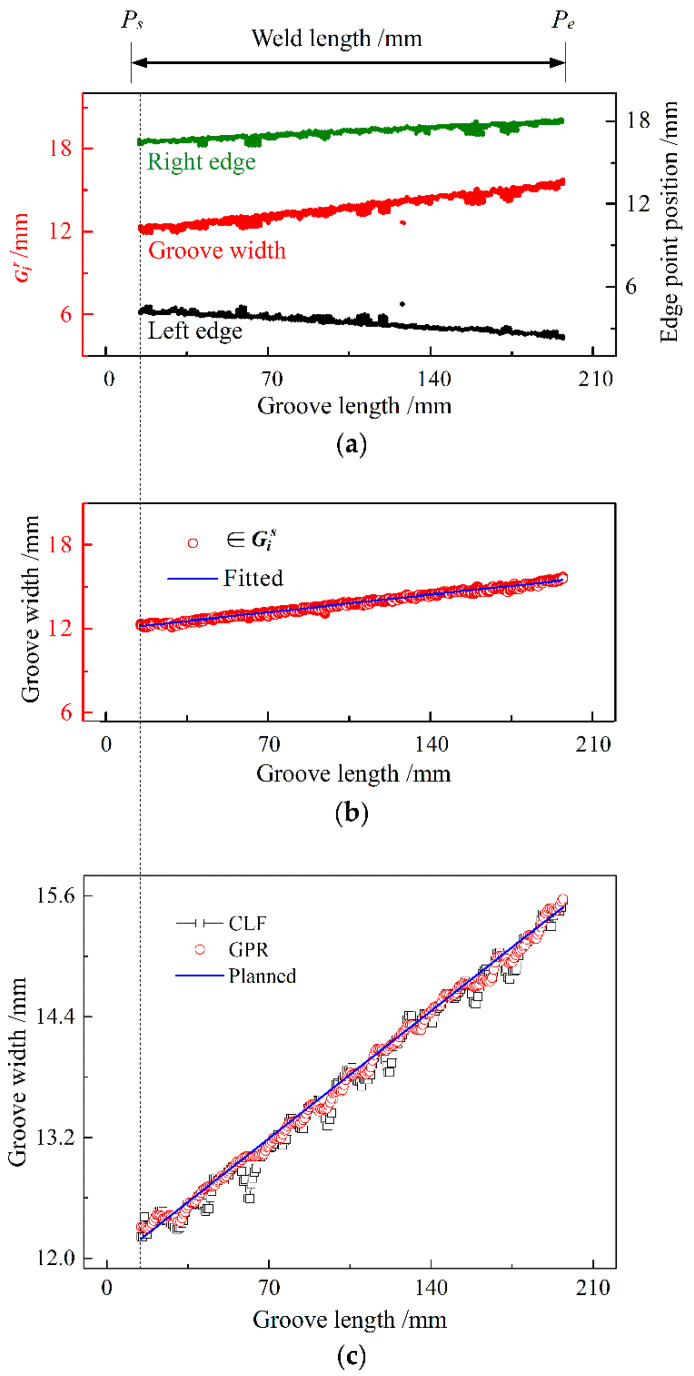
Detected results of width-varying groove: (**a**) edge point positions and instantaneous groove widths containing 269 sets of $G_{i}^{\;r}$; (**b**) true data belonging to $G_{i}^{\;s}$ and fitting line of the true data; (**c**) comparison of detected groove width values with the planned ones.

Figure 10 shows the detected results for a groove of varying width. In the experiment, an arc ignited within the groove ~10 mm away from the beginning of the groove, and the acquisition of welding images started after the arc was burning steadily. The arc was extinguished at an extinguishing point *P_e_* that was ~7 mm away from the groove terminal while finishing the collection of welding images. During the transiting period between the igniting point *P_s_* and the position of the dashed line in Figure 10, six frames of welding images were obtained to pre-determine the initial position of the ROI window. The detection of groove width then began from this dashed-line position.

To detect groove width, starting from the position of the dashed line, 270 frames of images in total were alternately intercepted by the ROI window for the left and right edges of the groove; that is, 135 images were collected for each of the left and right edges. Eighty instantaneous sampling positional values of corresponding groove edge points were calculated from each image to form each $G_{i}^{\;r}$, and two distribution graphs of all sampling values from these edge images are plotted for the left and right edge points of the groove by black and green solid points in Figure 10a, respectively. Accordingly, 269 raw data distribution matrices $G_{i}^{\;r}$ of groove width were obtained by a similar method to that used for the above constant-width groove, where each $G_{i}^{\;r}$ contains 80 instantaneous sampling values of groove width. Finally, the data in all sets of $G_{i}^{\;r}$ are indicated by red solid points in Figure 10a. During welding, several spatters occurred, which caused distinct changes in the instantaneous sampling values of the groove edge point position and groove width.

Similarly to the above constant-groove experiment, the ISDC algorithm eliminated false data from 269 sets of $G_{i}^{\;r}$, and accordingly, 269 true data distribution matrices $G_{i}^{\;s}$ were formed along the weld length. The true data in these matrices $G_{i}^{s}$ and their fitting line are indicated by red hollow circles and the blue line in Figure 10b, respectively. As a result, 269 detected values of *G_d_* were obtained through the GPR and CLF methods, as indicated by red hollow circles and black hollow squares in Figure 10c, respectively. Clearly, the detected values using the CLF method become low, around 61.68 mm, 122.20 mm and 159.60 mm of the groove length, owing to the occurrence of spatters and the resultant enhancing heredity effect of this mean filtering.

Compared with the planned values of groove width, as shown by the blue line in Figure 10c, the detection error of groove width ranges between −0.168 mm and +0.119 mm when using the GPR approach and between −0.447 mm and +0.196 mm when using the CLF method; the standard deviations of the detected values are 0.058 and 0.103, respectively, for the two methods, as listed in Table 4. These results indicate that the GPR approach can significantly reduce the interference effect of welding spatter and thus provide a higher detection precision of groove width than that obtained with the CLF method for a width-varying groove. Consequently, the accurate detection of groove width is achieved, which further demonstrates the effectiveness of the GPR approach. Moreover, the detection time of one groove width is ~30 ms for the GPR approach using a computer with a dominant frequency of 2.5 GHz, which can meet the requirement of future real-time control.

**Figure 11:**
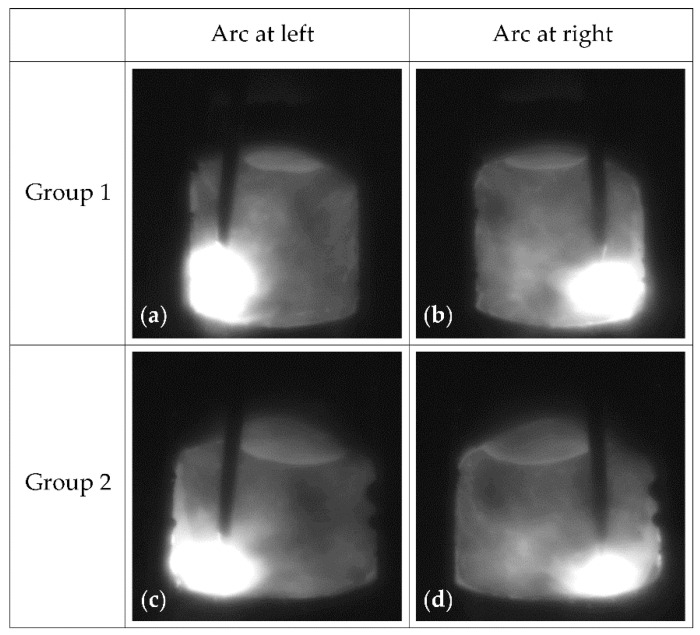
Actual welding images at groove lengths of around 29 mm and 179 mm: (**a**) welding image while arc stays at left at groove length of ~29 mm; (**b**) welding image while arc stays at right at groove length of ~29 mm; (**c**) welding image while arc stays at left at groove length of ~179 mm; (**d**) welding image while arc stays at right at groove length of ~179 mm.

Moreover, for a clearer understanding, two groups of welding images, which respectively correspond to groove lengths of around 29 mm and 179 mm in Figure 10, are presented in Figure 11. The two frames of images in each group were adjacent and captured at a time interval of ~200 ms. Accordingly, the four images were acquired at groove lengths of 29.04 mm, 29.72 mm, 178.64 mm and 179.32 mm, respectively, and the groove width grows from 12.45 to 15.17 mm for the two groups. Such an increase in groove width leads to a marked increase in the distances of the arc to the left and right sidewalls of the groove and thus actually results in the decreasing sidewall penetration of the groove.

### 5.4. Weld Formation Analysis

**Figure 12:**
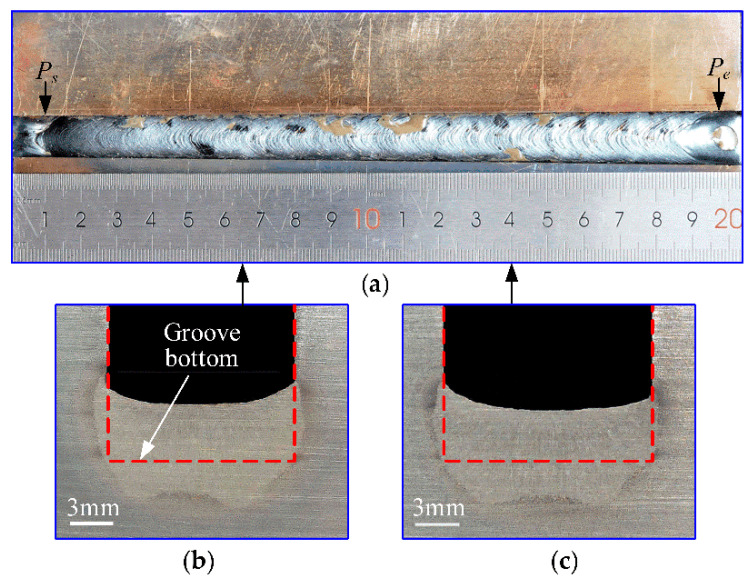
Photographs of weld formation in width-varying groove: (**a**) weld appearance; (**b**) bead cross-section at groove length of 65 mm; (**c**) bead cross-section at groove length of 140 mm.

As an example, Figure 12 shows photographs of weld bead formation for a width-varying groove, where *P_s_* and *P_e_* indicate the igniting and extinguishing points of the arc in the groove, respectively. Figure 12a shows the appearance of the weld surface, and Figure 12b,c indicate the cross-sections of the weld corresponding to the distances of 65 mm and 140 mm from the start of the groove, respectively. The bead cross-sectional photographs demonstrate that the weld did not fully penetrate the plate thickness, and thus, we do not supply the back photograph of the bead. At the two positions, the measured widths of the groove are 12.88 mm and 14.19 mm, respectively, and are reduced by 0.221 mm and 0.267 mm due to groove post-welding shrinkage, compared with the dynamic planned values of groove width in Figure 10. Therefore, this implies that the planned values are reasonable for the dynamic evaluation of the detected results.

Moreover, with an increase in groove width in the two cases, the average penetrations into the groove sidewall decrease from 0.872 to 0.654 mm; meanwhile, the height of the deposited metal, which is actually the vertical distance between the lowest position of the weld surface and the groove bottom, indicated by the red line in Figure 12b,c, declines from 3.777 to 3.317 mm. This suggests that, for the purpose of ensuring the consistency of sidewall penetration and weld height during welding, it will be necessary to adaptively control the swing angle of the arc and the volume of deposited metal according to the detected value of groove width in real time.

## 6. Conclusions

Based on infrared passive visual sensing, the detection approach and system of groove width were developed for swing arc narrow gap welding. This global pattern recognition approach can self-adaptively position the ROI window of groove edges, accurately extract the left and right edges of the groove on the opposite side of the arc by thresholding with four equal divisions and then detect the value of groove width by an in situ dynamic clustering algorithm based on dynamic classification. Experimental results show that the proposed approach has a strong ability to resist actual welding interferences and thus yields high detection accuracies, with detection error ranges of −0.086~+0.109 mm and −0.168~+0.119 mm for constant-width and width-varying grooves, respectively.

Based on the system and methods proposed, future work will include the concurrent control of the arc swinging angle and welding deposit so as to adapt to the probable variation in groove width. Accordingly, sidewall penetration will be kept sufficient and consistent by self-adaptive adjustment of the arc swinging angle, while a weld bead of constant height will be obtained by the self-adaptive regulation of welding deposit, finally building an intelligent and automatic system for the SA-NGW process.

## Figures and Tables

**Table 1:** Camera shooting conditions.

Parameter Name	Value
Central wavelength of narrowband filter (nm)	970
Neutral density filter (%)	30
Aperture	f/16
Exposure time (ms)	0.3
Shooting depression angle *θ* (°)	20
Global image size (pixels)	544 × 544

**Table 2:** Welding experimental conditions.

Parameter Name	Value
Average arc current (A)	302.5
Average arc voltage (V)	28.7
Arc current pulse frequency (Hz)	~222
Welding speed *V_w_* (mm s^−1^)	3.4
Solid wire diameter (mm)	1.2
Torch standoff height (mm)	20
Shielding gas/flowrate (L min^−1^)	Ar−20% CO_2_/25
Groove gap (mm)	14
Arc swing frequency (Hz)	2.5
Arc swing angle (°)	82
Arc at-sidewall staying time (s)	0.1
Conductive rod bending angle (°)	8

**Table 3:** Comparison of detection performance for constant-width groove.

Algorithm	Standard Deviation of Groove Edge Point Distribution	Size of Resistible Spatter	Error Range of Width Detection	Standard Deviation of Width Detection
GPR	1.449	≤2.19 mm	−0.086~+0.109 mm	0.035
CLF	4.581	≤0.58 mm	−0.465~+0.479 mm	0.115

**Table 4:** Comparison of detected results for width-varying groove.

Algorithm	Error Range (mm)	Standard Deviation
GPR	−0.168~+0.119	0.058
CLF	−0.447~+0.196	0.103

## Data Availability

Not applicable.
